# Mannan-Decorated Lipid Calcium Phosphate Nanoparticle Vaccine Increased the Antitumor Immune Response by Modulating the Tumor Microenvironment

**DOI:** 10.3390/jfb15080229

**Published:** 2024-08-16

**Authors:** Liusheng Wu, Lei Yang, Xinye Qian, Wang Hu, Shuang Wang, Jun Yan

**Affiliations:** 1 Center of Hepatobiliary Pancreatic Disease, Beijing Tsinghua Changgung Hospital, School of Medicine, Tsinghua University, Beijing 100084, China; wuliusheng852@126.com (L.W.); yangl23@mails.tsinghua.edu.cn (L.Y.); qianxy21@mails.tsinghua.edu.cn (X.Q.); w-hu21@mails.tsinghua.edu.cn (W.H.); s-wang22@mails.tsinghua.edu.cn (S.W.); 2 Yong Loo Lin School of Medicine, National University of Singapore, Singapore 19077, Singapore

**Keywords:** nanoparticle vaccine, antitumor immune response, calcium phosphate (CaP), tumor microenvironment, review

## Abstract

With the rapid development of tumor immunotherapy, nanoparticle vaccines have attracted much attention as potential therapeutic strategies. A systematic review and analysis must be carried out to investigate the effect of mannose modification on the immune response to nanoparticles in regulating the tumor microenvironment, as well as to explore its potential clinical application in tumor therapy. Despite the potential advantages of nanoparticle vaccines in immunotherapy, achieving an effective immune response in the tumor microenvironment remains a challenge. Tumor immune escape and the overexpression of immunosuppressive factors limit its clinical application. Therefore, our review explored how to intervene in the immunosuppressive mechanism in the tumor microenvironment through the use of mannan-decorated lipid calcium phosphate nanoparticle vaccines to improve the efficacy of immunotherapy in patients with tumors and to provide new ideas and strategies for the field of tumor therapy.

## 1. Introduction

Recently, tumor immunotherapy, as a revolutionary treatment, has brought new hope for patients with tumors [1,2,3]. However, despite some success, it still faces a number of challenges and limitations [4].

The core idea of tumor immunotherapy is to activate the body’s own immune system to attack and eliminate tumor cells [5]. However, the presence of the tumor microenvironment seriously affects the activity and function of immune cells, thus weakening the effectiveness of immunotherapy [6,7,8,9,10]. This microenvironment includes tumor cells, immune cells, blood vessels, interstitial cells, and other components, which interact with each other in a complex manner, thus resulting in immunosuppression [11]. The overexpression of immunosuppressive factors, the existence of immune escape mechanisms, and the immunosuppressive effect of tumor cells are some of the main challenges facing tumor immunotherapy [12]. To overcome these challenges, in recent years, scientists have focused on identifying new strategies and methods to improve the effectiveness of tumor immunotherapy [13,14,15,16]. As a new therapeutic strategy, nanoparticle vaccines have attracted much attention [17,18,19,20]. As a new nanoparticle carrier, the mannan-decorated lipid calcium phosphate nanoparticle vaccine has unique advantages and potential application prospects [21,22,23,24,25]. Mannose modification can make it easier for nanoparticles and tumor cells to be recognized and taken up [26]. This effect can increase the amount of vaccine that is enriched in tumor tissues, which improves the effectiveness of tumor immunotherapy [27,28,29,30]. In addition, mannose modification can also regulate the expression of immunosuppressive factors in the tumor microenvironment, destroy the interaction between tumor cells and immune cells, and further enhance the effect of immunotherapy [31]. We found that this nanoparticle vaccine can precisely target tumor cells and significantly improve the immune system’s ability to recognize and clear tumors by enhancing antigen delivery and immune cell activation. This study revealed how the vaccine, through a mannan-decorated strategy, regulates inflammation in the tumor microenvironment, inhibits immune escape mechanisms, and promotes the infiltration and activation of immune cells, thereby enhancing tumor-specific T-cell responses and cytotoxic activity [32]. The specific design of nanoparticles can optimize the efficiency of antigen delivery and improve the stability and biocompatibility of vaccines [33]. This reveals the potential of mannan-decorated lipid calcium–phosphorus nanoparticle vaccines as a novel immunotherapy strategy, providing an important theoretical and experimental basis for the development of more effective cancer immunotherapy protocols.

Our review systematically analyzes the research progress of tumor vaccines in enhancing the antitumor immune response and regulating the tumor microenvironment to provide a theoretical basis and practical guidance for further research in this field.

## 2. Regulation and Influence of the Tumor Microenvironment

The tumor microenvironment is an important aspect of tumor growth and development, and its characteristics are closely related to immunosuppressive mechanisms [34,35,36]. There are many immunosuppressive factors, such as transforming growth factor β (TGF-β) and interleukin-10 (IL-10), in the area around the tumor [35]. These factors can prevent immune cells from performing their functions and hinder their ability to find and kill tumor cells. Many immunosuppressant molecules, such as programmed death ligand-1 (PD-L1) and acidic extracellular matrix protein (TSP), are produced by tumor cells and surrounding cells [36,37,38,39,40]. These molecules interact with ligands on the surface of immune cells to allow the immune system to tolerate and escape. In addition, the highly acidified and hypoxic environment in the tumor microenvironment is also an important factor in immunosuppression, which not only affects the activity and function of immune cells but also induces apoptosis and functional abnormalities in immune cells [41,42,43,44]. The inflammatory response and immune cell infiltration in the tumor microenvironment are also closely related to immunosuppression [45,46,47,48]. The inflammatory response can promote the activation and infiltration of immune cells; however, it can also lead to their functional polarization and immune escape [49]. The tumor microenvironment provides favorable conditions for tumor escape by regulating the activity, function, and quantity of immune cells and changing the local physiological environment, thereby inhibiting the immune response [50].

**Figure 1:**
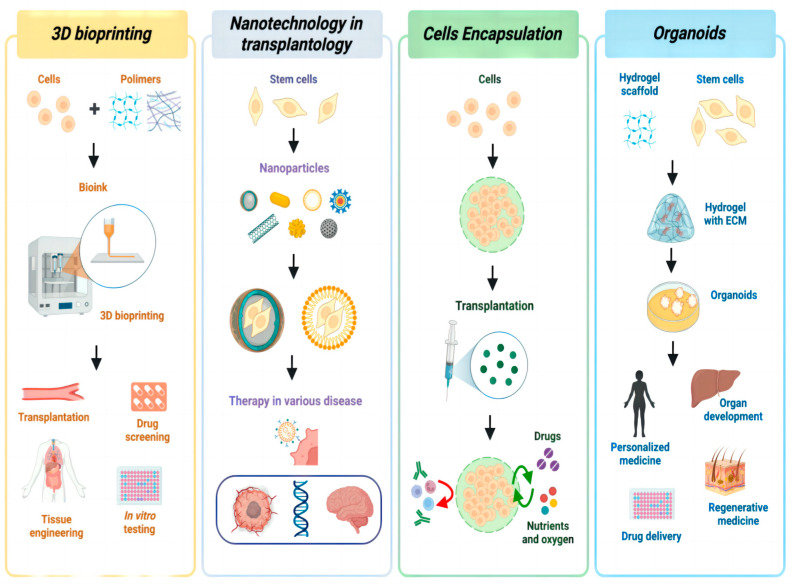
Three-dimensional bioprinting technology and the comprehensive nanocarrier application platform enable precise antitumor immunotherapy by combining biomaterials and nanotechnology. The platform uses bioprinting technology to accurately manufacture complex three-dimensional structures and load modified lipid calcium and phosphorus nanoparticles onto the vaccine, thereby enhancing the efficiency and specificity of antigen delivery, significantly regulating the tumor microenvironment, and enhancing the antitumor response of the immune system.

The combined application of 3D bioprinting technology and bio-nanocarrier technology has led to the construction of a new tumor treatment platform [51,52,53,54]. Three-dimensional bioprinting can accurately manufacture complex three-dimensional structures, whereas bio-nanocarrier technology can effectively deliver drugs or genes [55]. This combined application platform can enable customized tumor treatment programs, thus targeting drugs or gene carriers to the tumor site to improve treatment effectiveness [56]. In addition, the combination of these two technologies can improve the tumor immune microenvironment [57,58,59,60]. The immunosuppressive tumor microenvironment can be controlled by the release of nanocarriers carrying specific immunomodulators. It can also boost the activity of immune cells, help tumor cells die and immune cells invade, and improve the immune response of patients [61]. This combined application platform provides a new method for personalized and precise tumor therapy and has important clinical application prospects (Figure 1).

Cell interactions in the tumor microenvironment are closely related to those in the tumor mesenchyme and have important effects on the immune response [62,63,64]. The tumor stroma is composed of tumor cells, stromal cells, and stroma, and its complex cellular interactions affect the characteristics of the tumor microenvironment and the mechanism of immunosuppression [65]. Tumor cells influence the behavior of surrounding cells by secreting cytokines and chemokines, such as vascular endothelial growth factor (VEGF) and tumor necrosis factor (TNF), and by regulating tumor stromal formation and function [66]. Mesenchymal cells, including tumor-associated macrophages (TAMs) and cancer-associated fibrocytes (CAFs), interact with tumor cells by secreting cytokines and molecules, such as TGF-β and IL-6, to promote tumor growth, invasion, and metastasis and inhibit the activity of immune cells [67,68,69,70]. In a mouse model of brain metastases, studies have shown that the mannan-decorated lipid calcium–phosphorus nanoparticle vaccine has a significant targeted penetration ability and can successfully penetrate the meninges and accurately locate tumor cells.

**Figure 2:**
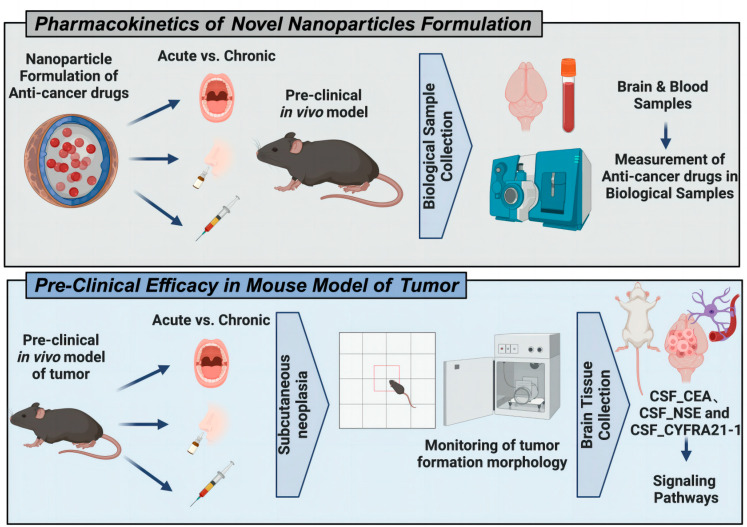
The nanoparticle carrier successfully penetrated the meninges and targeted and killed tumor cells in a mouse model of brain metastases. Through the modified lipid calcium and phosphorus nanoparticles, the carrier can efficiently deliver antitumor drugs, enhance the drug concentration at the tumor site, effectively destroy the tumor microenvironment, and promote the antitumor response of the immune system.

This nanoparticle carrier achieves effective drug delivery through specific binding to tumor cells, thus significantly killing tumor cells. More importantly, in this process, the nanoparticles not only directly act on the tumor cells but also regulate the interaction between the cells and the tumor matrix in the tumor microenvironment. The tumor microenvironment is composed of tumor cells, stromal cells, immune cells, and extracellular matrix, and its complex dynamic relationship plays a key role in the genesis and development of tumors [71]. By regulating this microenvironment, the mannan-decorated nanoparticles effectively destroy the interaction between tumor cells and stromal cells and weaken the viability and aggressiveness of tumor cells [72]. The nanoparticle vaccine not only works by directly killing tumor cells but also further enhances the antitumor immune response by modulating cell interactions in the tumor microenvironment (Figure 2).

Immune escape and tumor suppressor cells in the tumor microenvironment are important reasons for the hindered immune response [73,74,75]. Tumor cells and their surrounding cells and molecules work together in the tumor microenvironment to form a pattern of immune escape [76]. Tumor cells express excessive immunosuppressive molecules, such as PD-L1 and PD-L2, and immunosuppressive factors, such as TGF-β and IL-10. These factors prevent immune cells from functioning and hinder their ability to find and kill tumor cells [77]. In addition, tumor suppressor cells in the area around the tumor (such as TAMs and Tregs) control the immune response and help the tumor grow and spread by releasing immunosuppressive substances such as IL-10 and TGF-β [78,79,80].

Lipid calcium phosphate nanoparticles are usually prepared by the thin-film solution method, in which phospholipids and calcium phosphate are mixed in a certain proportion to form a lipid–calcium ion complex. Mannose-modified lipid calcium phosphate nanoparticles were formed by combining mannose with a lipid–calcium complex via the addition of an appropriate amount of mannose modifier.

In the tumor microenvironment, immune escape and tumor suppressor cells are key factors in tumor development and treatment difficulties. Tumor cells evade the surveillance and attack of the immune system through a variety of mechanisms, including regulating the activity of immunosuppressive cells such as regulatory T cells and myeloid suppressor cells, thereby suppressing the antitumor immune response. In addition, tumor suppressor cells in the tumor microenvironment promote tumor cell growth and metastasis by secreting a variety of cytokines and growth factors. It was found that the mannan-decorated lipid calcium–phosphorus nanoparticle vaccine can effectively regulate the tumor microenvironment and enhance the antitumor immune response, thus overcoming immune escape and inhibiting the function of tumor suppressor cells. Studies [81,82] in mouse models showed that the mannan-decorated lipid calcium–phosphorus nanoparticle vaccine was detected and monitored by photoacoustic imaging (PAI), demonstrating its distribution and biological distribution characteristics in vivo. Photoacoustic imaging technology combines the high contrast of optical imaging with the high resolution of acoustic imaging to monitor the distribution of nanoparticles in the body in real time without damaging tissues. This imaging technique demonstrated that mannan-decorated nanoparticles can effectively target tumor tissue and accumulate in the tumor microenvironment, thereby exerting their antitumor effects.

**Figure 3:**
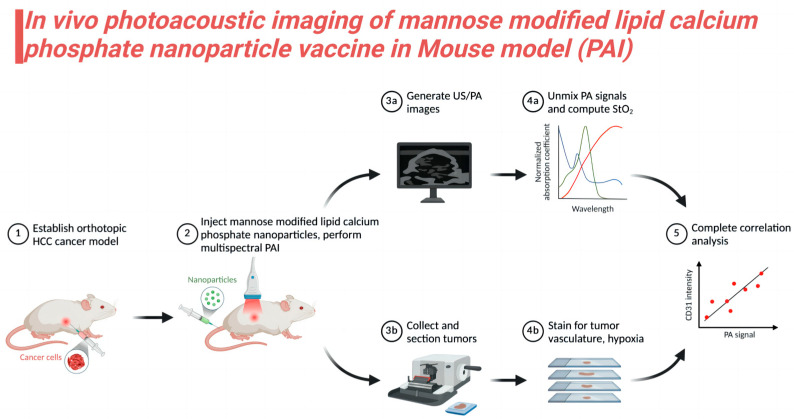
Photoacoustic imaging (PAI) of mannan-decorated lipid calcium–phosphorus nanoparticle vaccine in a mouse model. PAI technology uses its high resolution and deep imaging capabilities to clearly show the distribution and targeting effects of this nanoparticle in the body. Studies have shown that this vaccine can precisely accumulate in tumor tissue, significantly enhancing the immune system’s resistance to tumors through enhanced antigen delivery and regulation of the tumor microenvironment.

The accumulation of mannan-decorated nanoparticles at tumor sites can be clearly observed by photoacoustic imaging, which provides important support for the further understanding of its mechanism of action. These nanoparticles were not only able to directly kill tumor cells but also enhanced the antitumor immune response by modulating immune escape and tumor suppressor cells in the tumor microenvironment (Figure 3).

### 2.1. Role of Lipid Calcium Phosphate Nanoparticles in the Immune System

#### Lipid Calcium Phosphate Nanoparticles in the Immune System

Lipid calcium phosphate nanoparticles (100–200 nanometers, spherical) are important nanocarriers that have the potential to modulate antitumor immune responses in the immune system [83]. These nanoparticles are structurally designed to improve vaccine stability, biocompatibility, and immunogenicity. These nanoparticles can also mimic the structure and appearance of the virus. This strongly affects the immune system, which improves the body’s ability to find and destroy tumor cells [84,85,86,87]. In general, as an effective vaccine carrier, lipid calcium phosphate nanoparticles play an important role in the immune system, thus enhancing the antitumor immune response by promoting antigen presentation and immune cell activation and providing new strategies and hope for tumor treatment. mRNA LNPS (mRNA lipid nanoparticle) and calcium phosphate nanoparticles play different roles in vaccine delivery systems. mRNA LNPs are primarily used to deliver mRNA vaccines, and at their core are lipid nanoparticles that enclose mRNA molecules. This nanoparticle has a small particle size and good biocompatibility, which can effectively protect mRNA from degradation, improve its stability, and promote mRNA uptake in cells. In contrast, lipid calcium phosphate nanoparticles, with a calcium phosphate core and a surface modified with mannose to enhance targeting, are more commonly used to deliver proteins or antigens. In addition, the two have different application fields and characteristics. mRNA LNPs are widely used in the delivery of mRNA vaccines. Due to the easy degradation and instability of mRNA, LNPs can effectively wrap and protect mRNA and release it in the body to trigger an immune response. However, lipid calcium phosphate nanoparticles are more commonly used to deliver protein antigens, enhance targeting through mannan-decorated pathways, enter the tumor microenvironment, activate immune cells, and enhance the antitumor immune response.

**Figure 4:**
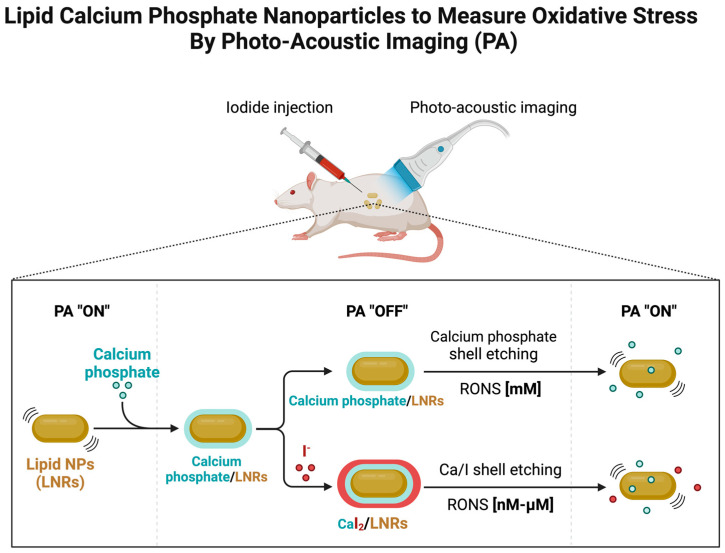
Schematic illustration of lipid calcium–phosphorus nanoparticles used to measure oxidative stress via photoacoustic imaging (PA). This schematic shows the mechanism of action of nanoparticles in vivo: mannan-decorated nanoparticles target tumor cells to monitor oxidative stress levels at tumor sites in real-time using photoacoustic imaging.

Our study used photoacoustic imaging (PA) to measure oxidative stress in lipid calcium phosphate nanoparticles. Lipid calcium phosphate nanoparticles were injected into the tumor site to locate the targeted organs and tumor sites in vivo, and the distribution and signal intensity of the lipid calcium phosphate nanoparticles were monitored in real time by using photoacoustic imaging technology; moreover, the intensity of the PA signal reflected the degree of oxidative stress [88]. During the observation process, we can infer the degree of oxidative stress in the tumor microenvironment from changes in signal intensity and further evaluate the role of lipid calcium phosphate nanoparticles in modulating the tumor immune response [89]. This process effectively combines lipid calcium phosphate, nanoparticle technology, and photoacoustic imaging technology to provide a feasible, noninvasive measurement method for the study of oxidative stress in the tumor microenvironment and provides an important reference for the optimal design of antitumor immunotherapy (Figure 4).

## 3. Design and Preparation of a Mannan-Decorated Lipid Calcium Phosphate Nanoparticle Vaccine

### 3.1. Effect of Mannose Modification on Vaccines

Mannose modification can confer good biocompatibility and immunological activity on nanoparticle vaccines [90,91,92,93,94,95,96,97,98,99,100,101,102,103,104,105,106,107,108,109,110,111,112,113,114,115,116,117,118,119,120,121]. Mannose modification can improve the stability of a vaccine and increase its circulation time in the body.

Mannose modification plays an important role in improving the stability of vaccines by covalently linking mannose to the surface of lipid calcium phosphate nanoparticles to form a protective film, which effectively prevents vaccines from being affected by the external environment in vivo. This protective film can resist adverse factors, such as enzyme degradation, pH changes, and temperature fluctuations, increasing the stability and reliability of the vaccine during storage and delivery. In addition, the addition of mannose can increase the duration of the vaccine cycle in the body. The modified nanoparticles have better biocompatibility and anti-clearance and can evade clearance by the liver or spleen, extending their residence time in the blood circulation.

In addition, mannan-decorated nanoparticles can bind specifically to immune cells to improve the cellular uptake rate and antigen delivery efficiency of the vaccine [122,123,124]. Mannose modification can also activate certain immune signaling pathways and improve the ability of antigen-presenting cells to express antigens, which strengthens the immune response of antigen-specific T cells [125,126,127,128]. During the design and preparation of the mannan-decorated lipid calcium phosphate nanoparticle vaccine, the influence of the mannose modification on the vaccine is reflected in its ability to improve its stability, enhance its immune activity, and promote antigen presentation, which provides strong technical support for tumor immunotherapy [129].

**Figure 5:**
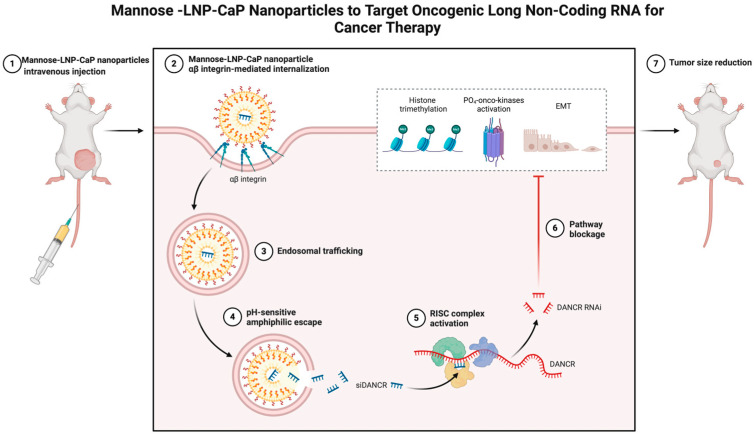
Schematic illustration of mannan-decorated lipid calcium–phosphorus nanoparticles (mannose-LNP-CaP) targeting carcinogenic long noncoding RNAs (lncrnas) for cancer therapy. This figure shows that mannan-decorated nanoparticles can specifically recognize and bind to cancer-causing lncrnas in tumor cells, inhibit their expression and function, and thus block the proliferation and metastasis of tumor cells.

Mannan-decorated lipid calcium phosphate nanoparticles have demonstrated a potentially revolutionary role in cancer therapy, and their ability to target cancer-causing long noncoding RNAs (ARAs) has brought new hope for cancer therapy [130]. By regulating the tumor microenvironment, nanoparticles can not only inhibit the growth and spread of tumor cells but also enhance the body’s antitumor immune response [131]. Moreover, combined with the research progress in tumor immunity, the results demonstrated that the use of mannan-decorated lipid calcium phosphate nanoparticles is not only a method of direct attack against tumor cells but also an innovative strategy for promoting the body’s immune system to participate in antitumor processes (Figure 5).

### 3.2. Design and Preparation of Lipid Calcium Phosphate Nanoparticles

#### 3.2.1. Preparation Method and Structural Advantages

Lipid calcium phosphate (CaP) nanoparticles have attracted much attention due to their unique advantages in vaccine delivery systems. Their design and preparation are essential for improving the bioavailability and immunological efficacy of vaccines [132,133,134,135]. Typically, the preparation process includes the solvent precipitation method and the coprecipitation method [136]. During solvent precipitation, the addition of phosphate and calcium ions causes the formation of calcium phosphate nanoparticles in solution. The coprecipitation of the drug and calcium phosphate is typically how the coprecipitation method produces the drug’s carrier [137,138,139,140,141,142,143,144,145,146,147,148,149,150]. The structural advantages provide a good platform for vaccine delivery and provide a foundation for regulating the tumor microenvironment and enhancing the antitumor immune response.

#### 3.2.2. Stability and Biocompatibility of Nanoparticles

Lipid calcium phosphate (CaP) nanoparticles are an important vaccine delivery system and have potential applications in antitumor immunotherapy [151]. In the design and preparation of these materials, we need to consider the stability and biocompatibility of the nanoparticles, which are essential for improving vaccine effectiveness and safety [152,153,154]. The stability of nanoparticles can be achieved by adjusting the preparation methods and adding surface modifiers. During the preparation process, the size, morphology, and dispersion of nanoparticles can be controlled via solvent precipitation or coprecipitation to ensure their stability [155]. Additionally, the use of appropriate surface modifiers, such as polyvinylpyrrolidone (PVP), can increase the stability of nanoparticles and prevent them from being cleared from the bloodstream and breaking down in living organisms [156].

Biocompatibility is an important indicator for evaluating the application of nanoparticles [157]. Mannan-decorated lipid calcium phosphate nanoparticles have received much attention due to their good biocompatibility [158]. Mannose, a natural sugar in the human body, has good biocompatibility and biodegradability and can reduce the immune response and toxic side-effects on the body [159]. Mannan-decorated nanoparticles can effectively avoid the clearance and decomposition of nanoparticles caused by immune responses, thus extending their circulation time in the body and increasing their accumulation in tumor tissues [160]. Additionally, changing the mannose concentration can improve the specific binding between nanoparticles and tumor cells, thus allowing for more precise targeted delivery and a better immune response against the tumor in the vaccine [161,162,163,164,165].

In general, the stability and biocompatibility of lipid calcium phosphate nanoparticles are problems that need to be considered and solved. Through rational design and preparation methods, as well as the introduction of biocompatible modifications such as mannose, the application of nanoparticles in antitumor immunotherapy can be effectively improved, thus providing strong support for regulating the tumor microenvironment and enhancing the antitumor immune response.

## 4. Immunomodulatory Mechanism of Mannan-Decorated Lipid Calcium Phosphate Nanoparticle Vaccine

### 4.1. Tumor Antigen Presentation and T-Cell Activation

#### 4.1.1. Mannan-Decorated Lipid Calcium Phosphate Nanoparticle Vaccine in Tumor Immunity

The mannan-decorated lipid calcium phosphate nanoparticle vaccine plays an important role in enhancing the antitumor immune response, and its immune regulatory mechanism involves several mechanisms [166]. As carriers, these nanoparticles can effectively load tumor antigens and their related immune stimulators (such as proteins and nucleic acids) in a stable manner on their surface or on the inside. Mannan-decorated nanoparticles can achieve precise, targeted delivery through specific binding to tumor cell surfaces [167,168,169,170]. This targeted loading allows the nanoparticles to be more efficiently sought out in tumor tissue and consumed by tumor cells [171]. NPs release tumor antigens that are loaded on themselves. This makes it easier for antigen-presenting cells, such as dendritic cells, to take in and process these antigens, which then causes immune cells to recognize and respond to the tumor antigens. In addition, mannose can interact with specific receptors on the surface of tumor cells to promote intracellular phagocytosis and the internal presentation of nanoparticles [172,173,174,175,176,177,178,179,180]. Finally, the release of these immune stimulators and the presentation of tumor antigens activate the body’s immune system, especially by promoting the activation and proliferation of antigen-specific T cells and B cells, thus strengthening the immune response to tumors [181,182,183,184].

The mannan-decorated lipid calcium phosphate nanoparticle vaccine regulates the tumor microenvironment and enhances the antitumor immune response by targeting tumor antigen delivery, promoting antigen presentation, and activating immune cells, thus providing new ideas and methods for tumor therapy.

#### 4.1.2. Activation of T Cells by a Mannan-Decorated Lipid Calcium Phosphate Nanoparticle Vaccine

The mannan-decorated lipid calcium phosphate nanoparticle vaccine changes the microenvironment of the tumor, which boosts the immune response against it [185]. One method of achieving this effect is by activating T cells; specifically, T cells are an important part of the immune system and play a key role in recognizing and eliminating tumor cells [186,187,188]. Mannan-decorated nanoparticles can enhance the immune response by promoting the activation and proliferation of T cells in a variety of ways [189].

Mannan-decorated nanoparticles can effectively improve the delivery efficiency of tumor antigens. These nanoparticles act as carriers that can stably load tumor antigens and release them into the tumor microenvironment [190]. Antigen-presenting cells (such as dendritic cells) take up and process these tumor antigens before presenting them to T cells and inducing an immune response to the tumor antigen. Mannan-decorated nanoparticles modulate immunosuppressive factors in the tumor microenvironment, thereby reducing T-cell suppression [191,192,193,194,195]. In the tumor microenvironment, the presence of immunosuppressive factors (such as PD-L1 and TGF-β) can inhibit the activation and function of T cells [196]. NPs modified with mannose can control the production and release of these immune-suppressing substances by interacting with specific receptors on the surface of tumor cells. This makes T cells less inhibited and more active, thus leading to increased cell growth and activation [197]. The mannan-decorated nanoparticles also activated T-cell costimulatory signaling pathways. Costimulatory signaling is a key factor in T-cell activation and proliferation, and the CD28/B7 and CD40/CD40L signaling pathways play important roles in T-cell activation and function [198,199,200]. NPs modified with mannose can activate these costimulatory signaling pathways by attaching to the correct receptors on the surface of T cells. This makes the T-cell immune response stronger.

**Figure 6:**
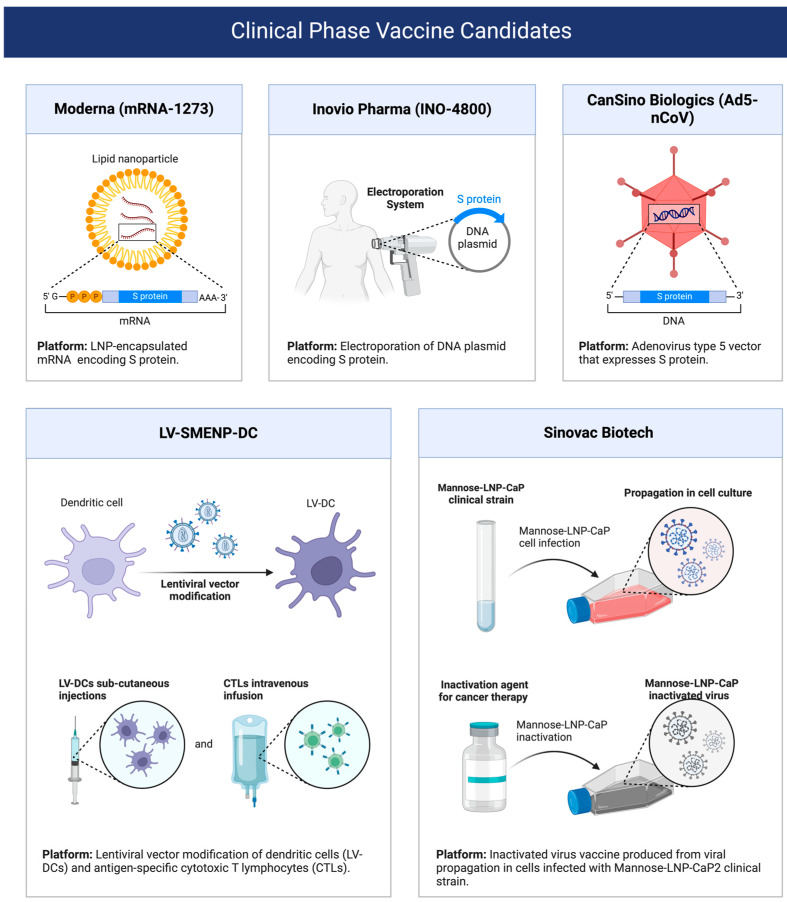
Summary diagram of clinical trial phase related to nanoparticle vaccine. This chart clearly shows the various stages of nanoparticle vaccine development from the early stage to clinical application, including preliminary safety evaluation (Phase I), dose optimization and immune response analysis (Phase II), large-scale multi-center efficacy and safety validation (Phase III), and continuous post-marketing monitoring and efficacy evaluation (Phase IV).

The use of a mannan-decorated lipid calcium phosphate nanoparticle vaccine, which is an innovative immunotherapy method, has received extensive attention and research in recent years [201]. By modulating the tumor microenvironment, this vaccine can significantly enhance the antitumor immune response, thus providing new possibilities for tumor treatment. Several studies [202,203,204,205] have explored the treatment of this nanoparticle nucleic acid vaccine through clinical trials. These clinical trials typically involve the treatment of tumor patients in groups, with one receiving the mannan-decorated lipid calcium phosphate nanoparticle vaccine and the other receiving either standard treatment or a placebo. The main purpose of the trial was to assess the effect of the vaccine on tumor growth in patients and the extent to which it activated the immune system [206]. By comparing the effects of treatment on different groups of patients, researchers can assess the effectiveness and safety of the vaccine. In clinical trials [207,208,209,210,211,212], researchers typically examine data on several aspects, including changes in tumor size, longer patient survival, and increased immune cell activity. These data can not only help determine the therapeutic effect of the vaccine but also provide an important basis for further optimization of the vaccine design and treatment plan (Figure 6).

### 4.2. Enhancement in the Tumor Immune Response and Establishment of Immune Memory

The use of a mannan-decorated lipid calcium phosphate nanoparticle vaccine is a novel tumor immunotherapy method that can enhance the immune response to tumors by regulating the tumor microenvironment [213]. Previous studies [214,215,216,217,218,219,220] have shown that vaccines can activate the body’s immune system, promote the expression and recognition of tumor-associated antigens, and trigger a specific immune response against tumor cells. Through mannose modification, the vaccine can be more effectively taken up by antigen-presenting cells and improve the efficiency of antigen delivery in the lymph nodes, thus further activating immune cells such as dendritic cells and T cells and enhancing the potential of the immune response [221].

In the establishment of immune memory, the application of a vaccine has also shown remarkable results [222,223,224,225]. After inoculation with mannan-decorated lipid calcium phosphate nanoparticles, the body can form a long-term memory of tumor antigens [226]. This immune memory allows the body to recognize and clear tumor cells quickly and efficiently during subsequent tumor invasion, thereby reducing the risk of tumor recurrence and metastasis. In addition, the establishment of immune memory also provides a solid foundation for subsequent immunotherapy, thus enabling the body to produce a more durable and powerful response to further treatment with tumor vaccines or other immunomodulators [227,228,229,230].

**Figure 7:**
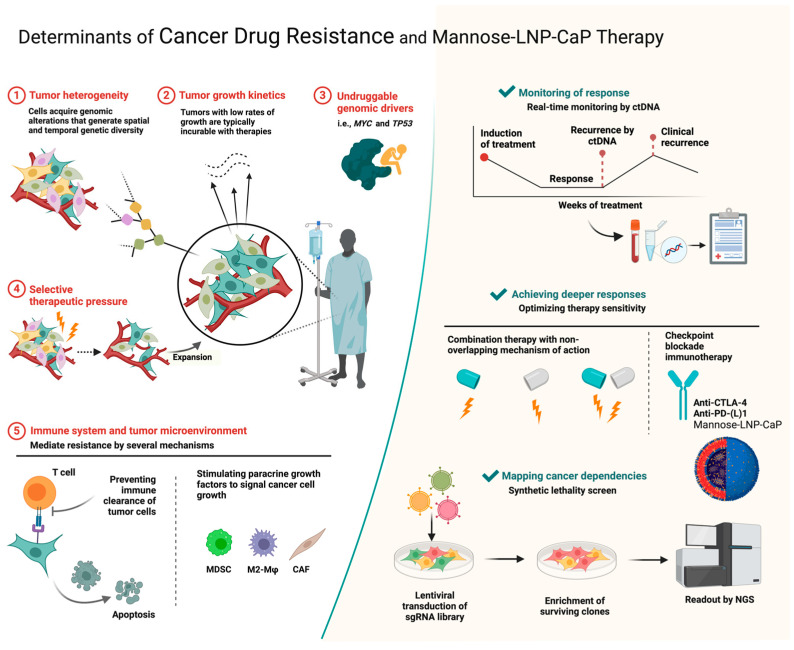
Drug resistance factors in cancer and treatment of mannose-modified lipid calcium and phosphorus nanoparticles (mannose-LNP-CAP). This figure shows that cancer drug resistance includes key factors such as the complexity of the tumor microenvironment, gene mutations, and drug efflux pumps. Mannose-LNP-CaP therapy overcomes drug resistance by targeting these resistance mechanisms, especially by precisely regulating the tumor microenvironment, enhancing antigen delivery, and activating immune cells, and significantly improves the efficacy of antitumor immune responses.

As a new method to treat tumors with immunotherapy, a mannan-decorated lipid calcium phosphate nanoparticle vaccine has shown great promise in improving the immune response to tumors and building immune memory [231,232,233,234,235]. This provides new ideas and strategies for the future treatment of cancer and is expected to play an important role in clinical practice, thus resulting in more effective treatments and a better quality of life for patients [236,237,238,239,240]. The main determinants of drug resistance include tumor microenvironment heterogeneity, immunosuppressive mechanisms, and inefficient drug delivery [241]. A mannan-decorated lipid calcium phosphate nanoparticle vaccine can improve the immunogenicity of tumor cells, regulate the tumor microenvironment, and promote an antitumor immune response by stimulating natural antigen presentation (Figure 7).

### 4.3. Analysis of Immune Cell Infiltration in Tumor Tissue

Tumor tissue immune cell infiltration is an important indicator for evaluating the ability of the mannan-decorated lipid calcium phosphate nanoparticle vaccine to enhance the antitumor immune response by regulating the tumor microenvironment [242]. The infiltration of different types of immune cells (such as CD8+ T cells, CD4+ T cells, and natural killer cells) in tumor tissues can be quantitatively analyzed via immunohistochemical staining, flow cytometry, and other techniques. It was found that the mannan-decorated lipid calcium phosphate nanoparticle vaccine can significantly increase the amount of CD8+ T-cell infiltration in tumor tissues, improve the ratio of CD8+/CD4+ T cells, and promote the transformation of the tumor immune microenvironment [243,244,245]. In addition, the vaccine can also effectively increase the degree of invasion of natural killer cells, thereby enhancing the clearance of tumor cells [246]. An analysis of tumor immune cell infiltration showed that a mannan-decorated lipid calcium phosphate nanoparticle vaccine could significantly regulate the tumor microenvironment and enhance the antitumor immune response.

## 5. Promising Research Prospects for Preclinical Research

As a novel tumor immunotherapy strategy, the use of a mannan-decorated lipid calcium phosphate nanoparticle vaccine has shown great potential in preclinical studies [247,248,249,250]. Through in-depth investigation of its mechanism of action, we found that the vaccine can effectively regulate the tumor microenvironment and enhance the antitumor immune response of the body. Previous studies [251,252,253] have shown that mannan-decorated nanoparticles can promote the uptake and endocytosis of tumor cells through specific targeting, thereby improving the efficiency of antigen delivery and activating the activity of tumor-associated antigen-specific T cells. In addition, the vaccine can also induce immune cells in the tumor microenvironment, such as plasma cells and dendritic cells, to release proinflammatory factors and inhibit the function of immunosuppressive cells, thereby promoting the activation and expansion of T cells, enhancing the killing ability of cytotoxic T lymphocytes and ultimately realizing the effective elimination of tumors.

## 6. Discussion

In future studies, we can further optimize the formulation and preparation process of a mannan-decorated lipid calcium phosphate nanoparticle vaccine to improve its stability and bioavailability in vivo, thereby enhancing its antitumor immunotherapy effect [254]. In addition, vaccines could be explored in combination with other tumor therapies, such as chemotherapy, radiotherapy, and immune checkpoint inhibitors, to achieve better therapeutic outcomes. In addition, it is possible to design personalized treatment regimens for different types and stages of tumors and verify their safety and efficacy through preclinical and clinical studies. In general, mannan-decorated lipid calcium phosphate nanoparticle vaccines have broad application prospects in the field of tumor immunotherapy and are expected to become an important strategy for tumor therapy in the future.

In comparison with existing therapies and the literature, mannan-decorated lipid calcium–phosphorus nanoparticle vaccines show significant advantages and unique mechanisms of action [255]. Traditional cancer treatments such as surgery, radiation, and chemotherapy, although effective in some cases, are often accompanied by a higher risk of side-effects and tumor recurrence. In addition, although many immunotherapies have demonstrated effectiveness against specific cancer types, their universality and targeting are still insufficient, and they are prone to immune escape and adverse reactions. In contrast, mannan-decorated nanoparticle vaccines achieve more efficient antigen delivery and specific immune responses by precisely regulating the tumor microenvironment, resulting in more durable and intense antitumor effects in mouse models. A variety of nanoparticle vaccines in the existing literature have shown some antitumor potential, but most of them lack the regulatory ability to target the tumor microenvironment, which plays a key role in tumor growth and immune escape. The nanoparticle vaccine in this review not only improves tumor targeting and immune cell recognition through mannose modification but also significantly enhances the effect of the antitumor immune response by regulating the activity of immune cells in the tumor microenvironment, inhibiting the tumor-related inflammatory response and reducing the immunosuppressive mechanism.

## 7. Conclusions

MK-modified NPs can effectively regulate the tumor microenvironment, inhibit tumor growth, and enhance the infiltration of immune cells. The mannan-decorated lipid calcium phosphate nanoparticle vaccine showed good potential for regulating the tumor microenvironment, promoting immune cell infiltration, and inducing antibody and T-cell responses, thus providing new ideas and strategies for tumor immunotherapy.

## Data Availability

Not applicable.
